# The Lymph Node Microenvironment May Invigorate Cancer Cells With Enhanced Metastatic Capacities

**DOI:** 10.3389/fonc.2022.816506

**Published:** 2022-02-28

**Authors:** Tianhang Li, Tianyao Liu, Zihan Zhao, Xinyan Xu, Shoubin Zhan, Shengkai Zhou, Ning Jiang, Wenjie Zhu, Rui Sun, Fayun Wei, Baofu Feng, Hongqian Guo, Rong Yang

**Affiliations:** ^1^ Department of Urology, Affiliated Drum Tower Hospital, Medical School of Nanjing University, Nanjing, China; ^2^ Jiangsu Engineering Research Center for MicroRNA Biology and Biotechnology, State Key Laboratory of Pharmaceutical Biotechnology, School of Life Sciences, Nanjing University, Nanjing, China; ^3^ Nanjing Drum Tower Hospital Clinical College of Jiangsu University, Nanjing, China

**Keywords:** lymph node, tumor evolution, epigenetic reprogramming, immune escape, PD-L1, stemness, tumor metastasis

## Abstract

Cancer metastasis, a typical malignant biological behavior involving the distant migration of tumor cells from the primary site to other organs, contributed majorly to cancer-related deaths of patients. Although constant efforts have been paid by researchers to elucidate the mechanisms of cancer metastasis, we are still far away from the definite answer. Recently, emerging evidence demonstrated that cancer metastasis is a continuous coevolutionary process mediated by the interactions between tumor cells and the host organ microenvironment, and epigenetic reprogramming of metastatic cancer cells may confer them with stronger metastatic capacities. The lymph node served as the first metastatic niche for many types of cancer, and the appearance of lymph node metastasis predicted poor prognosis. Importantly, multiple immune cells and stromal cells station and linger in the lymph nodes, which constitutes the complexity of the lymph node microenvironment. The active cross talk between cancer cells and immune cells could happen unceasingly within the metastatic environment of lymph nodes. Of note, diverse immune cells have been found to participate in the formation of malignant properties of tumor, including stemness and immune escape. Based on these available evidence and data, we hypothesize that the metastatic microenvironment of lymph nodes could drive cancer cells to metastasize to further organs through epigenetic mechanisms.

## Introduction

Lymph node metastasis served as the most common metastatic manner for many tumor types, such as bladder cancer, breast cancer, melanoma, and colorectal cancer (1–4). In many cases, the lymphatic system was the first receiving station for the metastatic tumor cells, and the presence of lymph node metastasis has been recognized as an effective predictor for the further metastasis to other organs and the poor prognosis of cancer patients (5, 6). With regard to the mechanisms of lymph node metastasis, multiple studies have been conducted to demonstrate that the properties of lymph node metastasis for some cancers were related to the upregulation of lymphangiogenic growth factors, higher lymphatic invasiveness, and increased density of lymphatic vessels (7, 8). In addition, due to the massive enrichment of diverse immune cells within the lymph nodes, the active or suppressive immune status of the lymph node microenvironment is also found to mediate the elimination or protection of cancer cells within the metastatic niche of lymph nodes (9–11). However, a definite theory to elucidate the mechanism of lymph node metastasis remains unclear.

In recent years, the rapid innovation and progress of tumor lineage tracking technology and cancer sequencing techniques enabled us to reconstruct and detect the phylogenetic and evolutionary process of the distinct clonal populations of cancer cells. Multiple studies have demonstrated that metastases are derived from distinct clonal groups from the primary tumor, including “monoclonal seeding” and “polyclonal seeding.” Tumor heterogeneity is originated from continuous mutations produced mainly during cell division, and the subsequent formation of multiple subpopulations provided the raw material for the secondary environmental selection. A comparative analysis performed on the whole-exome sequence expressed between paired primary tumor and metastasis samples showed that the clonal selection on a major subpopulation from the primary tumor is more limited to a single major clone and harbored fewer metastasis-driven specific mutations than expected across several cancer types (12). However, this clone-selective pressure could be amplified by treatment, which resulted in evolved drug resistance instead of metastasis capacities (13). Nevertheless, these observations are based on retrospective analysis on the static gene expression profile, which largely limited the resolution and accuracy. To overcome this problem, Simeonov et al. developed a novel single-cell pedigree localization and tracking approach based on CRISPR-Cas9 technology to reveal the evolutionary history of single-cell transcriptional states during the process of metastasis in pancreatic cancer (14). Surprisingly, they found that over half of the clones of the primary cancer cells were confined to the primary sites and never metastasize to other sites, while the dominant clones survived in the metastatic sites with an enhanced expression of epithelial-to-mesenchymal transition (EMT) state. In consistency with previous studies, this research emphasized the domination of advantageous clonal selection in the metastasis. More importantly, it pointed out the distinct genetic expression phenotype associated with the highly metastatic cancer cells, which brought us with several new questions: (1) Do the genetic mutations related with the metastatic capacities all derive from the evolutional selection pressure during metastasis? (2) Could the microenvironment of the metastasis sites exert regulatory functions on the disseminated cancer cells to endow them with enhanced malignant biological characteristics? (3) What factors determine the tropism of further metastasis of tumor, and is the dissemination to different organs totally randomized or programmed?

Following these questions, we found quite a few related studies revealing somewhat different answers. In contrast with the mainstream cognition that primary lesion is the dominant origin for further dissemination of metastatic cells to other organs, a range of studies indicated that most seeding of metastatic cells originated from metastatic sites rather than the primary tumor (15, 16), which suggested that the clonal selection from primary tumor may be insufficient to explain the mechanism of cancer metastasis and the cross-seeding between metastases and secondary metastasis from distinct organs may have underestimated functions on the regulation of metastatic cancer cells.

Recently, Zhang et al. utilized the approach of parabiosis and an evolving barcode system to trace the evolution history of metastasis in mouse models of prostate and breast cancer, which are all highly bone-metastatic cancers. Interestingly, they also detected that majority of the metastases in further organs were from the first metastatic bone lesions rather than the primary tumor (17). Of note, this study demonstrated that the bone microenvironment not only simply served as an early transport station for metastatic cells but also exerted crucial epigenetic modulatory functions on the stemness of the cancer cells, which finally enhanced the metastatic capacities of cancer cells and promoted the further dissemination to further organs.

In summary, the process of metastasis is not only simply a unidirectional evolution route under the selective pressure of different metastatic environments, but rather an active two-way interactive process, which finally contributed to the evolution and further dissemination of highly metastatic cancer cell populations. Notably, the microenvironment of the metastatic sites could initiate a process of epigenetic reprogramming on the cancer cells, which explained the enhanced metastatic capacities and increased mortality after the event of first metastasis. As has been mentioned before, the lymph node is the first metastatic organ with the greatest possibility for many cancers, such as bladder cancer and gastric cancer. Correspondingly, the higher possibility of further dissemination and a poorer prognosis are found to be significantly associated with lymph node metastasis. However, there still lacks an exact explanation for this troublesome clinical problem.

## The Hypothesis/Theory

We proposed that in those cancers tending to first metastasize to the lymph nodes, the microenvironment of lymph nodes would facilitate the cancer cells to undergo secondary metastasis to multiple further organs through epigenetic regulation or other mechanisms, which most likely mediated the enhancement of the immune-escape ability of tumor cells. In particular, the cross talk between cancer cells and the lymph node metastasis served as a crucial and indispensable part of the tumor evolution during the process of metastasis.

## Justification of the Hypothesis

### Cancers With Lymph Node Involvement Are More Progressive and Malignant

Multiple studies verified that lymph node metastasis was an independent predictor for the oncological progression and patient survival in many cancers (1, 18–20). Of note, the probability of metastasis into other further organs significantly increased along with the appearance of lymph node metastasis. One of the reasons may be the route of lymphatic vessels facilitates the migration of cancer cells, which accelerated the speed of tumor dissemination. However, there still existed several problems remaining to be resolved. For one thing, under the circumstance of cancer with distant metastases, we are still unclear about the major origin of the metastatic cells in the distant organs. According to traditional understanding, the hematogenous metastasis from the primary tumor should be the “root of all evils” for the distant metastasis. However, recent studies demonstrated the high heterogeneity of the tumor population during the process of metastasis, which indicated the complexity of distribution of tumor cells and their cloning clustering (14–17). Also, emerging evidence proved that the major origin of further metastatic tumor cells on distant organs may not necessarily be the primary tumor, but rather the first metastatic lesions, such as bones (17). More importantly, the microenvironment of the bones enhanced the stemness of tumor cells through epigenetic modulation, which finally contributed to the further metastasis (17). To some extent, the lymph node shares many similar characteristics with bone for tumor metastasis, such serving the most frequent first metastatic niche for multiple cancers and increased probabilities of further metastasis. Taking these factors into consideration, we may naturally come to the hypothesis that the lymph node could also exert crucial functions on the metastatic tumor cells to enhance their metastatic abilities for further dissemination. To elucidate this issue, we may firstly trace the evolutionary process and cloning distribution in the context of lymph node metastasis. Once we find that most clones of tumor cells in the further distant organs are derived from the lymph node metastases rather than the primary tumor, it may be more solid evidence for our hypothesis.

## Classical Immune Phenotype Alterations of Metastatic Cells in the Microenvironment of Lymph Nodes

**Figure 1:**
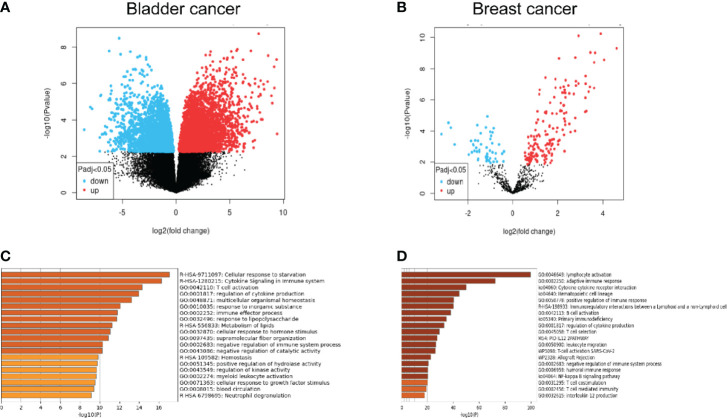
**(A, B)** Comparative analysis from the microarray data extracted from the GEO database illustrating the significantly upregulated and downregulated genes between lymph node metastases and primary tumor. **(C, D)** Gene-enrichment analysis showing the predicted enriched pathways mediated by the significantly upregulated genes in the lymph node metastases compared with the primary tumor using Metascape (http://metascape.org/).

As the most widely distributed immune organ in our body, lymph nodes have unique environmental characteristics. Traditionally, lymph nodes were viewed as killers against invaded cancer cells. Resident or recruited cytotoxic immune cells, such as natural killer cells, CD8+ T cells, and M1-type macrophages, would help eliminate tumor cells through different mechanisms (21–23). However, the fact of high incidence of lymph node metastasis indicated that metastatic tumor cells as well as the microenvironment of the lymph node must encounter significant alterations to help the tumor cells escape the immune attack from immune cells. Recent studies have demonstrated that in the tumor-involved lymph nodes, a range of immune-suppressive cells and biological factors would be recruited or activated, which facilitated the survival and growth of metastatic tumor cells. The mechanisms include the decrease of CD8+ T cells, myeloid-derived suppressor cell (MDSC) expansion, and upregulation of regulatory T cells (24–27). However, the exact alterations of metastatic tumor cells from primary tumor cells remained unclear. Some studies presented changes in expressive levels of immune-checkpoint proteins such as PD-1 and PD-L1 of the lymph node microenvironment and explored its correlations with prognosis (28–30), but these studies are unable to show the changes in tumor cells themselves. To explore the alterations in genetics as well as the biological phenotypes between the primary tumor and the lymphatic metastases, we performed a bioinformatic analysis to compare the differential genes and the enrichment of possible pathways from the Gene Expression Omnibus (GEO) database. The differentiated expressed genes between primary tumor and lymph node metastases were extracted from GSE121738 for bladder cancer and GSE180186 for breast cancer (**Figure 1**). Surprisingly, we found that the predicted enriched pathways were focused on the negative regulation of immune responses, which revealed that the metastatic cells in the lymph node possessed upregulated immune-escape characteristics (31). From this result, we may naturally speculate that those metastatic cells colonized on the lymph nodes could strive to survive and resist the immune attack from lymph nodes through acquisition of higher immune-escape capacities. More importantly, in this way, these metastatic cells would possess stronger progressive phenotypes, which facilitates them to metastasize into further organs. The possible immune-escape mechanisms may include upregulation of immune-checkpoint proteins, such as PD-L1 and CD47, expression and release of immune-suppressive cytokines or chemokines, such as TGF-β1, IL-10 and so on, and abnormal regulation of non-coding RNA components. Nevertheless, we need to elucidate the origin of the acquisition and whether strengthening of these immune-escape abilities is from the natural cloning selection during metastasis or from the phenotypic regulation of the lymph node environment on cells or both.

## Complexity and Heterogeneity of the Lymph Node Microenvironment

In our hypothesis, the complexity of the lymph node microenvironment and the heterogeneity of its composed cell populations are the potential inducers or regulators for cancer second-time evolution with enhanced metastatic capacities. Therefore, it is essential to carefully review the structural and compositional properties of lymph nodes, especially in the context of cancer involvement.

Three main structural elements, including the cortex, paracortex, and medulla, help constitute the anatomical framework of the lymph node, and different types of immune cell populations own their main regional distribution. The cortex, consisting of the follicle, the interfollicular zone, and the subcapsular sinus, is the main resident area for B cell and dendritic cells, which regulated the process of antigen presentation and antibody production, while the paracortex is mainly distributed with T cells and the medulla is covered with macrophages (32, 33).

Generally, immune cells are the “flesh” of the lymph node and the stromal cells are the “skeleton.” Functionally, lymph node stromal cells not only help form the architecture of the lymph node but also play important roles in regulating the normal function of those immune cells mainly by releasing cytokines such as CCL19, CCL21, and IL17 (33, 34). Similar with previously mentioned immune cells, lymph node stromal cells also possess strong heterogeneity and regional distribution difference. Based on the surface biomarker expression and regional location, the lymph node stromal cells could be mainly divided into four subtypes, including lymphatic endothelial cells, blood endothelial cells, fibroblastic reticular cells, and CD31gp38-double negative cells (35).

Besides the cellular component of the lymph node microenvironment, the extracellular matrix is an indispensable constitute for the lymph node. Structural stability and biological sustainability are ensured by the complex network of the lymph node extracellular matrix (36).

To conclude, the lymph node microenvironment is a very complex network involving various cell types and components. However, it is the great cellular heterogeneity and the structural complexity that provide the metastatic cancer cells with more possibilities of multidirectional genetic selective pressure and more opportunities of cell–cell interactions. In the next section, we will discuss several potential mechanisms of regulatory roles for reprogramming metastatic cancers cells in the lymph node microenvironment, especially based on the possible origin from the distinct component of the lymph node.

## Potential Mechanisms of Regulatory Roles for Reprogramming Metastatic Cancer Cells in the Lymph Node Microenvironment

Herein, we listed several potential possible regulatory mechanisms which may explain the enhancement of metastatic capacities of cancer cells granted by the microenvironment.

### Potential Regulatory Roles Played by Lymph Node Stromal Cells

As has been discussed in the previous section, lymph node stromal cells not only served as major structural participators in the microenvironment of lymph node but also exerted important immune-regulatory functions on the immune cells. A previous study revealed that the lymphatic endothelial cells are the major source to produce sphingosine-1-phosphate (S1P) to mediate the emigration of lymphocytes from the lymph node (37, 38). However, as a pleiotropic bioactive molecule, S1P is involved in multiple pathological processes, including carcinogenesis. Genetic expression could be regulated by S1P through histone deacetylase binding, which may lead to abnormal expression of a series of genes such as p21 (39). Whether this epigenetic modulation could be utilized by lymphatic endothelial cells to regulate cancer cell phenotype alteration warrants further investigations. Also, proliferation capabilities of a range of tumor cells have been reported to be upregulated *via* the S1P–S1PR pathway (40–42). In addition, invasiveness, which is another key factor for metastasis, has also been found to be enhanced by S1P activation in glioma, which drove the malignant behavior (43). What determines our attention is that S1P-exerted functions are limited not only on the cancer cells but also on the whole immune environment, including M2 differentiation (44, 45) and Treg accumulation (46), and these immunosuppressive cells may also play regulatory roles for cancer cells, which indicated that lymphatic endothelial cells may also reprogram cancer cells through indirect mechanisms.

Under inflammatory conditions, nitrite oxide (NO) was found to be produced by lymphatic endothelial cells and fibroblastic reticular cells as an immune-suppressive pattern to inhibit the overactivation of T cells in the lymph node (47, 48). However, this pathway could also be hijacked by metastatic cancer cells for self-evolution. As a powerful deamination agent, NO was found to cause DNA damage through C–T transformation, which brought great genotoxicity and negatively influenced the DNA repair ability (49). This way, naturally we may hypothesize that the metastatic cancer cells arriving in the lymph node would face greater possibilities of potential gene mutations, which would provide a large-scale gene screening pool under the selective pressure of the lymph node microenvironment and more possibly evolve into a more malignant phenotype. Notably, different concentration levels of NO may reprogram the cancer cells through different oncogenic mechanisms (50). Epigenetic modulations could also be imposed on the cancer cells by the stromal cell-secreted NO. The DNA methylation event and histone acetylation event are the two major epigenetic regulatory mechanisms for cancer cells (51, 52). What is interesting is that NO was proved to be able to increase acetyl events through enhanced production of acetyl CoA induced by deprivation of aconitase 2 (53). Also, the methylation level of DNA CpG islands could be upregulated under the condition with high levels of NO through a direct inhibition of demethylase (54). In all, epigenetic alterations on the cancer cells could be regulated by the lymph node stromal cells. Recent studies have indicated that many crucial oncogenic pathways are mediated by these epigenetic mechanisms such as immune escape ability (55, 56) and stemness (57, 58). However, whether similar epigenetic reprogramming processes would happen in the lymph node microenvironment to drive the enhancement of cancer cell malignancy *via* NO produced by lymph node stromal cells remains to be elucidated.

When we mentioned the term “lymph node stromal cells” under the context of cancer metastasis, it is natural for us to associate another well-known term, cancer-associated fibroblasts (CAFs), which represents a unique stromal cell subpopulation, originated from preexisting normal stromal cells, which coevolved with the cancer cells in the tumor microenvironment with a common pro-tumor capability (59, 60). Similarly, the lymph node stromal cells seemed to also share these characteristics when they encountered the arrival of metastatic cancer cells. Nevertheless, existing investigations still cannot prove that the stromal cells in the metastatic lymph node micro-environment experienced similar reprogramming. But we may easily infer that a great possibility does exist that the lymph node stromal cells may coevolve with the cancer cells and finally acquire a phenotype similar with CAFs to in turn exert crucial regulatory functions on the cancer cells to facilitate their further invasion and metastasis. However, this issue required further investigations to verify.

### Potential Regulatory Roles Played by Lymph Node Immune Cells

As has been discussed before, in addition to the lymph node stromal cells, immune cells are the other important cell population in the lymph node microenvironment. The immune cells mainly include two subpopulations: myeloid cells and lymphoid cells. The myeloid cells are composed of dendritic cells and macrophages while the lymphoid cells include B cells and T cells (22, 61). During the process of lymph node metastasis, innate and adaptive immune responses would be activated for tumor elimination while immune escape could also be induced to facilitate cancer progression. Combining the results of the gene-pathway enrichment analysis above and the common phenomenon of cancer immune escape in the lymph node, we assume that the immune cells in the lymph node may participate in the regulation of the malignancy enhancement of metastatic cancer cells.

PD-L1 is a classical immune-checkpoint protein mainly expressed on the cell membrane of tumor cells. By interacting with PD-1, which is expressed on T cells, PD-L1 induced the apoptosis and dysfunction of T cells and suppressed the anticancer immunity (62, 63). In consistency with our previous findings, many studies have demonstrated that lymph node metastasis in multiple cancers possessed overexpression of PD-L1. In addition, the high expression level of PD-L1 in lymph nodes strongly correlated with the poor prognosis of cancer patients (28, 64, 65). The mechanisms controlling PD-L1 expression covered different levels including genomic alterations, transcription, post-transcription, translation, and post-translation. Cha et al. made a brilliant review summarizing the intricate regulatory networks of PD-L1 expression (66). Aberrant inflammatory pathways have been clarified to contribute to loss of immunosurveillance in the tumor microenvironment, including transcriptionally upregulating the PD-L1 expression. MYC is a well-studied oncogenic transcription factor, which has been found to be able to bind the PD-L1 promoter and subsequently promote PD-L1 mRNA expression (67), while in the microenvironment of lymph node metastases, emerging evidence indicates that MYC expression showed an abnormal upregulation in the metastatic lymph nodes (68–70). Therefore, it is possible that the lymph node microenvironment harnessed specific mechanisms to promote PD-L1 expression of cancer cells through MYC modulation.

As an important part of the immune system, the lymph nodes basically assumed two major rules: one is to eliminate the dangerous foreign “non-self”, and the other is to recognize the “self” and avoid the unnecessary harm to the host. In the context of cancer, interferon-γ (IFN-γ) plays a central role against cancer development with its cytotoxic, pro-apoptotic, and immune-boosting functions. However, in the face of immune attack, tumor cells are able to hijack the IFN-γ pathway to enhance the PD-L1 expression (66, 71, 72), which finally help tumor cells survive the immune killing and survive. Considering the large enrichment of killer immune cells in the lymph node metastasis, metastatic cancer cells would encounter fierce attack in the metastatic niche, which offered great opportunity for them to evolve in this harsh living environment, in part through the upregulation of PD-L1 membrane expression. Moreover, other inflammatory cytokines also participated in the induction of mRNA expression of PD-L1, including TGF-β, TNF-α, and IL-6 (66, 73–75), and these cytokines are also important participators in the lymph node metastases, which may also serve as alternative pathways for the upregulation of PD-L1 in cancer cells and train them to become more powerful metastatic cancer cells for further dissemination in other organs. Seeing that the major origin of these inflammatory mediators is the immune effector cells, we assumed that the PD-L1 upregulation under the pressure of immune attack from immune cells of the lymph node may be a crucial step for the enhancement of immune-escape ability of metastatic cancer cells. However, this hypothesis has not been reproduced or confirmed in previous studies and we still need further experiments to verify this possibility. Also, the upregulation of PD-L1 as well as other immune-checkpoint proteins may not only be dependent on the direct defense mechanism for the cytotoxic immune cells but also derive from an indirect regulatory direction from other immune-suppressive cells in the microenvironment of lymph nodes. Multiple studies suggested that the presence of a high infiltration of Treg cells in the lymph node is significantly associated with cancer lymph node metastasis (76–78). As a powerful subpopulation of immune-suppressive cells, Treg cells would produce immune-suppressive cytokines, such as IL-10 and TGF-β, for controlling the balance of immune function (79, 80). However, these immune-suppressive mediators could also be utilized by metastatic cancer cells for their self-reinforcement. TGF-β and IL-10 could all serve as upstream activation factors for PD-L1 expression (66, 75). Similar functions may also be observed in other immune-suppressive cells, such as M2 macrophages and MDSCs, *via* future investigations. In a word, during the process of reprogramming for metastatic cancer cells, the driving forces may come from two directions: the direct pro-tumor regulations from immune-suppressive cells and the resisting self-defense mechanisms against the immune-killing cytotoxic cells. This regulatory process could be simultaneous or sequential, but we assume that the difference of strong and weak is dependent on the degree of metastasis.

In addition to the upregulation of membrane immune-checkpoint proteins, the metastatic capacities of cancer cells may also depend on the epigenetic reprogramming of other key genetic targets. TGF-β was identified as a powerful oncogenic cytokine mainly produced and released by tumor cells in the tumor microenvironment (81, 82). For one thing, TGF-β is able to promote Treg cell differentiation and recruitment, suppression of CD8+ T cell function, and MDSC expansion, which focused on the formation of the immune-suppressive microenvironment (83–85). For another, TGF-β could directly promote tumorigenesis through induction of epithelial to mesenchymal transition (EMT) and angiogenesis (86, 87), which both led to easier and faster tumor metastasis. Notably, a higher expression of TGF-β1 in the lymph node is significantly associated with the more malignant phenotype and poor survival across a range of cancer types (88–90). Then, we may naturally question whether the interactions between the lymph node microenvironment and metastatic cancer cells could enhance the regulatory function of TGF-β1 in metastatic cancer cells. MicroRNAs (miRNA) are well-studied small non-coding RNA molecules widely distributed in our body (91). By recognizing and binding with the target mRNA with the complementary sequences, miRNA could then repress the transcription of the specific RNA, which plays major regulatory roles in various physiologic or pathologic processes, including oncology (92, 93). For example, the decrease of the miR-200 family by TGF-β could promote TGF-β expression in return, which formed a positive feedback loop to further enhance the oncogenic function of TGF-β (94, 95). Importantly, a range of crucial tumor-suppressor miRNAs, including the miR-200 family, was also found to show a dramatic downregulation in the triple-negative breast cancer type with lymph node metastasis (96), which indicated that the aberrant expression of specific miRNAs was significantly associated with the enhanced metastatic capacities of cancer cells. Nevertheless, whether the miRNA networks regulate the enhancement of metastatic capabilities of cancer through modulating the TGF-β pathway remained elusive.

### Potential Regulatory Roles Played by Other Eenvironmental Factors of the Lymph Node Microenvironment

As has been discussed above, non-coding RNAs (ncRNAs), including miRNA, circular RNAs (circRNA), long non-coding RNAs (lincRNA), and tRNA-derived small RNAs (tsRNA), are pivotal biological molecules exerting powerful epigenetic functions majorly controlling the post-transcription of a range of crucial genes during the process of carcinogenesis (97–99). Multiple studies have demonstrated that tumor cells could influence the lymph node microenvironment through secretion of individual or exosome-capsuled ncRNAs, which further facilitate their dissemination and progression (100–102). However, in the tumor-involved lymph node microenvironment, the ncRNA expression profile of the lymph node stromal cells and immune cells is still unclear to us. Up until now, a series of circular miRNAs, such as miR-20a, miR-203 (103), and miR-10b (104), have been identified as potential biomarkers for lymph node metastasis prediction. However, their exact origin and biological functions are still unknown. Chen et al. performed a comprehensive bioinformatic analysis based on a comparative miRNA array between lymph node metastases and paired primary tumor and found that miR-10a was significantly upregulated in the lymph nodes with a further verification by detection of relative cell lines (105). Then, we may question whether ncRNA secretion from the immune cells or stromal cells into the metastatic cancer cells could also epigenetically regulate their phenotype alterations and further result in their stronger progressive malignancy. Further studies are required *via* a finer cell clustering before gene sequencing for the lymph node metastasis, which may indicate the alterations of the ncRNA expressive profile under the condition of tumor involvement and further verify the reaction from the lymph node microenvironment to the cancer cells.

Hypoxia represented a specific environmental status with a low oxygen pressure of less than 5–10 mmHg (106). In the context of cancer lymph node metastasis, hypoxia has been found to be a factor that cannot be neglected for driving progression and metastasis (107, 108). The reason for the formation of hypoxia mainly originated from the oxygen overconsumption and enhanced metabolism under the pressure of tumor overgrowth. Importantly, hypoxia along with its induced alterations of a series of key oncogenes is an indispensable contributory factor for lymphangiogenesis (109, 110). HIF-1α, which is the major functional effector in response to hypoxia, could regulate the remodeling of lymph node endothelial cells (111, 112). Nevertheless, HIF-1α could also exert key regulatory functions on the cancer cells. Multiple studies suggested that HIF-1α served as a key transcriptionally regulatory element for PD-L1 upregulation in a range of cancers (66, 113–115). Therefore, great possibilities exist that metastatic cancer cells that colonized on the involved lymph nodes may acquire enhanced immune-escape capacities in response to the hypoxia status in the lymph node through the HIF-1α pathway. Of note, hypoxia and HIF-1α pathway-induced phenotype alterations not only are limited in PD-L1 expression but also may influence other important characteristics and functions of cancer, such as EMT (116, 117) and glycolysis metabolism induction (118, 119).

In conclusion, due to the complexity of the components in the lymph node and the rich changes in face of the tumor involvement, various subpopulations of the cells and other environmental elements of the lymph node microenvironment own the potential to modulate or reprogram the metastatic cancer cells. The enhanced metastatic capability conferred by the lymph node microenvironment may not only depend on the induction of immune escape but also may derive from the other key oncogenic pathway activation and strengthening, such as stronger tumor stemness and migratory and invasive abilities. However, considering the distinct environmental characteristics of the lymph node, we may prefer the hypothesis that the enhanced metastatic capabilities conferred by the lymph node microenvironment largely relied on the stronger immune-escape ability. The possible regulatory roles played by different components of the lymph node microenvironment are summarized in **Table 1**.

**Table 1:** Possible regulatory roles played by different components from the lymph node microenvironment for metastatic tumor reprogramming.

Regulators	Possible regulatory mechanisms	Obtained malignant phenotypes	References
Lymphatic endothelial cell	S1P–S1PR interaction	Enhanced proliferative capabilities; enhanced invasiveness	(37, 38, 40–43)
Lymphatic endothelial cell; fibroblastic reticular cell	NO-induced gene instability and mutations; NO-mediated epigenetic modulations	Increased probability and directions for evolution; stronger cell population heterogeneity; increased stemness and immune-escape ability	(47–49, 51–54)
Cytotoxic T cell	IFN-γ, TNF-α, IL-6 induced immune-checkpoint protein expression pathway; PD-L1 upregulation	Increased immune-escape ability	(66, 71–75)
Regulatory T cell; MDSC; M2 macrophage	TGF-β, IL-10-induced immune-suppressive pathways; induction of EMT	Increased immune-escape ability; enhanced migratory ability	(66, 75–78)
Altered expressive profile of ncRNAs	Epigenetic regulations of key oncogenes	Stronger progressive phenotype	(100–105)
Hypoxia	HIF-1α induced PD-L1 upregulation; EMT enhancement; glycolytic propensity regulation	Increased immune-escape ability; stronger survivability	(111, 112, 116–119)

## Discussion

Traditionally, tumor metastasis into further organs is identified as the terminal step of tumor progression, which served as the major cause of cancer-related death. Rapid development of gene-editing and sequencing technology allowed us to trace the metastasis process more precisely. Growing evidence suggested that tumor metastasis is not a unidirectional and even process originating from the parental niche but a cross-seeding interactive process between multiple metastatic environments. Importantly, further secondary metastasis within further organs is found to be more homologous with the first metastatic niche rather than the primary tumor, and the microenvironment of the first metastatic environment could exert epigenetic reprogramming function on the cancer cells, which strengthened their metastatic capabilities and facilitate them to further metastasize. Lymph node metastasis is commonly viewed as a crucial factor of poor prognosis in cancer patients and served as the first metastatic niche for many tumor types. The unique state of immune cell enrichment and inflammatory status determined that potential training effects could be performed on the cancer cells, involving upregulation of immune-escape capacities. Thus, we hypothesize that in those cancers tending to metastasize to lymph nodes first, the microenvironment of metastatic lymph nodes could invigorate the cancer cells with enhanced metastatic abilities, which contributed to the further metastasis.

As we discussed before, the contributory driving forces for possible reprogramming regulatory mechanisms on the malignancy of metastatic cancer cells could be mainly divided into three branches. The first one is from the lymph node stromal cells. Under normal physiologic conditions, these stromal cells are identified as structural supporters as well as regulators or recruiters of immune cells (33, 120, 121). Nevertheless, their possible epigenetic modulatory roles on the metastatic cancer cells should not be ignored. For one thing, NO, as a powerful genotoxic substance, could be secreted by lymphatic endothelial cells and fibroblastic reticular cells (47, 48), which may create an environment for more possibilities of gene mutations and epigenetic reprogramming in cancer cells. The increased cell heterogeneity provided primitive accumulation of evolutionary materials while the epigenetic modifications offered the required opening and closing of key genes. For another, the S1P–S1PR pathway could be activated by lymphatic endothelial cells to promote the enhancement of proliferative and invasive capabilities of cancer cells, which may explain the reason for the strengthening of second-metastatic ability (40–42). Notably, we presented a possibility that the lymph node stromal cells could experience a phenotype alteration under the invasion of cancer cells, which may form a positive-feedback loop to lead to their further negative regulation on the cancer cells.

The second possible regulatory pathway is originated from the immune cells in the lymph node microenvironment. As is shown in **Figure 1**, the upregulated genes in lymph node metastatic cells compared with the primary niche demonstrated significant enrichment in a range of immune-related pathways, including the negative regulation of the immune system process, T cell activation, and immune effector process. Considering the highly immune-attack pressure in the lymph node, it is reasonable to hypothesize that the cancer cells may acquire the enhancement of immune-escape capabilities to adapt to or resist the immune killing from the lymph node immune cells and further metastasize into further organs. PD-L1 has been recognized as a significant biomarker for lymph node metastasis in a range of cancers (28, 64, 65). What is intriguing is that the functional cytokines and inflammatory mediators produced by those immune-killing cells may be utilized by cancer cells as upstream factors to in turn upregulate the expression of PD-L1 (66, 71–74). In addition, the infiltration of immune-suppressive cells, such as Treg cell, MDSC, and M2 macrophage, could also exert similar functions (66, 75, 79, 80).

The third possible regulator we hypothesized is the environmental characteristic of hypoxia in the lymph-node microenvironment. HIF-1α, in response to the oxygen concentration in the lymph node microenvironment, could serve as an important transcriptionally regulatory mediator for a series of key phenotypes in the cancer cells, such as immune escape (113–115), EMT (116, 117), and metabolism alteration (118, 119).

**Figure 2:**
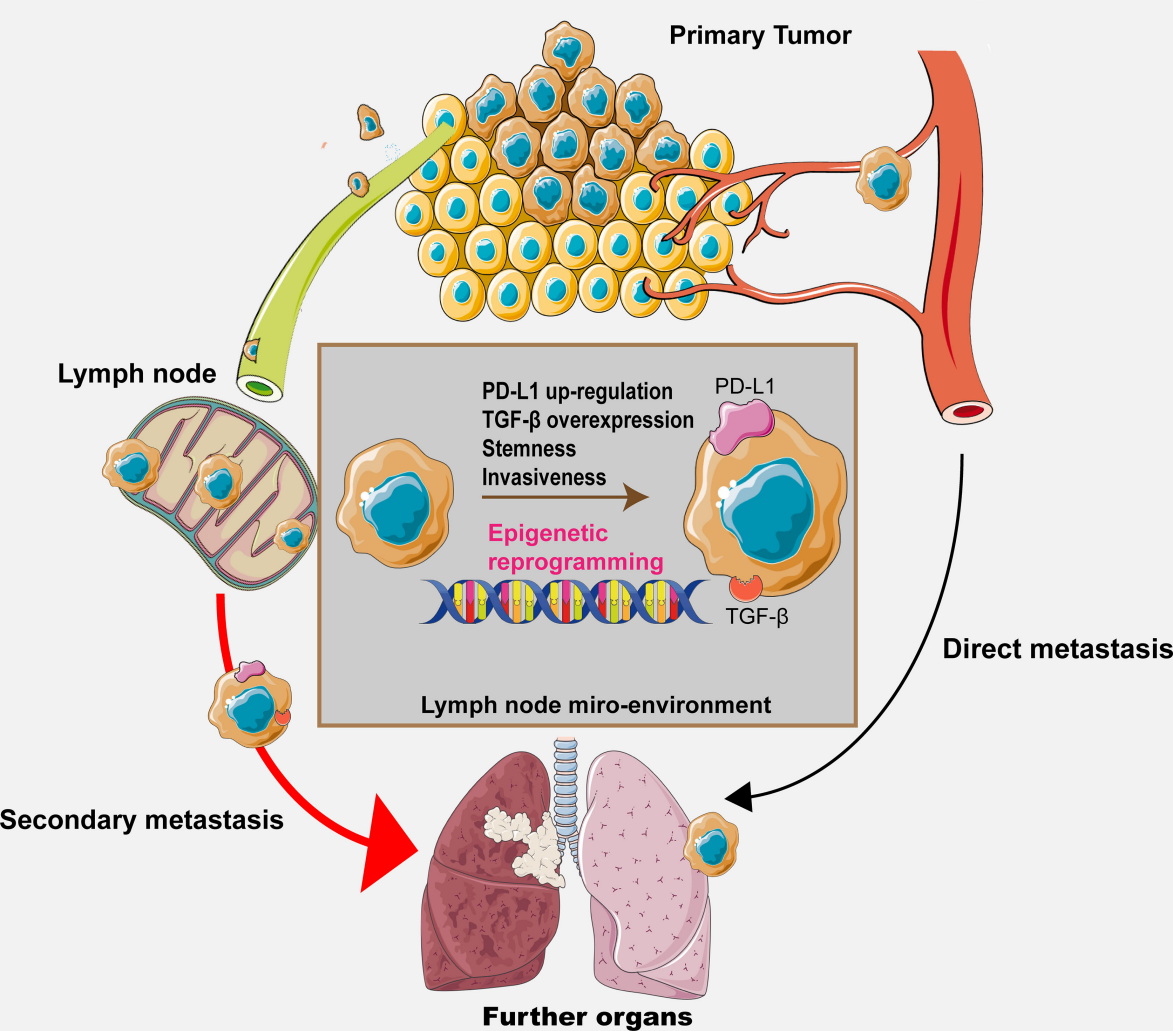
The graphical hypothesis illustrating the regulatory process and potential mechanism throughout the metastasis process.

Despite that a few specific alterations along with their regulators in the lymph node microenvironment have been discussed and hypothesized previously, it is certain that we still cannot include all the possible regulatory pathways because the co-evolutional process between the metastatic cancer cells and the lymph node microenvironment involves constant and complex cell–cell interactions. Therefore, further investigations are required for elucidating the exact mechanisms. The hypothesis is shown in **Figure 2**, illustrating the whole process and potential mechanisms.

## Data Availability Statement

The original contributions presented in the study are included in the article/supplementary material. Further inquiries can be directed to the corresponding authors.

## Author Contributions

RY and HG designed and constructed the scientific idea. ThL extended and systemically analyzed the feasibility of the scientific hypothesis and wrote the paper. TyL, XX, and ZZ participated in the discussion and provided the crucial components of this hypothesis. SbZ and SkZ participated in the discussion and collected the data. NJ, WZ, and RS polished the language and revised the manuscript. FW and BF participated in the discussion and drew the figures. All authors contributed to the article and approved the submitted version.

## Funding

This work was supported by the National Natural Science Foundation of China (81772727, 81772710 and 82172691) and Nanjing Science and Technology Development Key Project (YKK19011).

## Conflict of Interest

The authors declare that the research was conducted in the absence of any commercial or financial relationships that could be construed as a potential conflict of interest.

## Publisher’s Note

All claims expressed in this article are solely those of the authors and do not necessarily represent those of their affiliated organizations, or those of the publisher, the editors and the reviewers. Any product that may be evaluated in this article, or claim that may be made by its manufacturer, is not guaranteed or endorsed by the publisher.
